# Comparison of Anion-Exchange Membranes for Diffusion Dialysis of Mixtures of Acids and Their Iron Salts

**DOI:** 10.3390/membranes14010006

**Published:** 2023-12-24

**Authors:** Helena Bendová, Libor Dušek, Jiří Palarčík

**Affiliations:** Institute of Environmental and Chemical Engineering, Faculty of Chemical Technology, University of Pardubice, 53210 Pardubice, Czech Republic; libor.dusek@upce.cz (L.D.); jiri.palarcik@upce.cz (J.P.)

**Keywords:** continuous diffusion dialysis, hydrochloric, nitric, hydrofluoric acid, ferric salt, anion-exchange membrane

## Abstract

This study presents the possibility of using diffusion dialysis for the separation of inorganic acids (hydrochloric, nitric, and hydrofluoric) and their ferric salts whose composition corresponds to that of real spent pickling solutions. At a steady state, the transport properties of three different anion-exchange membranes (Fumasep-FAD, Neosepta-AFN, and Neosepta-AHA) are compared using a continuous counter-current dialyzer. At a constant composition of the solutions (acid concentration 3 mol L^−1^ and iron concentration 30–40 g L^−1^), the effects of volumetric liquid flow rates on the transport rate of H^+^ and Fe^3+^ ions through the membrane are studied. The dialysis process is characterized by the recovery of acids and the rejection of salts. Furthermore, the values of the dialysis coefficients of acids, iron, and the acid/iron separation factors are calculated and compared. The volumetric flow rates of the inlet streams change in limits from 3 × 10^−8^ to 6 × 10^−8^ m^3^ s^−1^ (from 3 to 6 L h^−1^ m^−2^, relative to the membrane area). A comparison of the tested membranes shows slightly better results for acid recovery, iron rejection, and acid/iron separation factors for the Fumasep-FAD membrane than for the Neosepta-AFN membrane. However, the results obtained show that both of these anion-exchange membranes can be considered good separators for tested mixtures that simulate real spent pickling solutions, and there is a good precondition for using diffusion dialysis for processing these solutions in industrial practice. On the contrary, very low values of acid recovery and the overall dialysis coefficient of acid are found for the Neosepta-AHA membrane in the test range of the volumetric flow rate, and, thus, this membrane is insufficient for the adequate separation of these acids and iron salts.

## 1. Introduction

It is wellknown that large amounts of spent acidic fluids containing toxic metal ion complexes are generated during several processes of the mining, metallurgical, metal-processing, and nuclear-fuel-reprocessing industries, including pickling, leaching, etching, electroplating, and metal-refining industries, which cause a serious hazard to the living and non-living environments. Among the technologies that enable at least a partial regeneration of these effluents are the use of ion exchangers, evaporation, crystallization, metal extraction using suitable solvents, pyrometallurgical methods, and membrane separation processes (diffusion dialysis, membrane distillation, and electrodialysis) [1]. Diffusion dialysis using anion exchange membranes driven by the concentration gradient is considered an effective technology with a low energy consumption and little environmental pollution. The disadvantage of diffusion dialysis is that the flow of the substance through the membrane is relatively small compared to membrane separation processes in which an electric field is a driving force (e.g., in electrodialysis) [2].

In recent years, several studies have been carried out on the regeneration of acid waste liquids by diffusion dialysis [3,4,5,6,7,8,9,10,11,12,13,14,15]. In the literature [3], diffusion dialysis was used for the treatment of spent desoldering solutions with the content of tin, iron, copper, and lead in HNO_3_. Wang et al. [4] recovered sulfuric acid from a stone coal acid leaching solution by diffusion dialysis. In the literature [5], diffusion dialysis was coupled with precipitation cementation to separate and recover nitric acid, Cu^2+^, Zn^2+^, and Pb^2+^ from wastewater from a brass pickling bath. Bendova and Weidlich [6] separated nickel from the spent Raney Ni catalyst by continuous diffusion dialysis with the Neosepta-AFN membrane.

Gueccia et al. [7] separated the mixture of hydrochloric acid and iron and zinc from highly concentrated pickling solutions using a batch dialyzer and a larger continuous dialyzer, both with the Fumasep-FAD membrane. In their next work, an innovative membrane process was designed that combines diffusion dialysis and membrane distillation technologies with a reactive precipitation unit to recover acid from the pickling solution [8], and a pilot operation of this combined process was also presented [9]. In [10], authors performed an economic analysis of this developed process.

Zhang et al. [11] proposed a pressure-concentration diffusion dialysis process to overcome the limitations of diffusion dialysis, such as low processing capacity and water osmosis. A combined hybrid membrane process of diffusion dialysis and electrodialysis was used to recycle sulfuric acid from the spent copper plating solution (containing FeSO_4_, and CuSO_4_) [12] to treat battery recycling wastewater containing H_2_SO_4_ and NiSO_4_ [13] or to recover acids (hydrochloric, nitric, and sulfuric) from metallurgical acid wastewater containing Fe^n+^ salts [14]. In [15], a counter-current dialyzer with a Neosepta-AFN membrane was used to separate the model mixture of hydrofluoric acid and ferric nitrate and a real spent pickling solution.

To determine the characteristics of diffusion dialysis, two types of devices are used: a batch cell and a continuous dialyzer. Batch dialysis was used, for example, for the separation of an aqueous solution of HCl + FeCl_2_ [16] or for the separation of an HNO_3_ + Fe(NO_3_)_3_ mixture [17] using an anion-exchange membrane Neosepta-AFN. In the case of continuous processes, the most widely used type of membrane modules is the flat plate dialyzer [3,4,5,6,7,8,9,10,11,12,13,14,15]; however, spiral wound diffusion dialysis membrane modules have attracted much attention recently. A spiral wound module with the Fumasep-FAD membrane was, e.g., used to separate H_2_SO_4_ and Cu^2+^ and Fe^2+^ salts [18] and to separate HCl and Zn^2+^, Cr^3+^, Ni^2+^, and Fe^2+^ salts [19]. In [20], a tubular bag membrane submerged in a matrix liquid was used to separate the H_2_SO_4_/FeSO_4_ solution through a batch or semi-continuous diffusion dialysis process.

Attention is also paid to the comparison of membranes for diffusion dialysis. The authors of [21] present a comparative study of different types of ion-exchange membranes (heterogeneous cation and anion exchange membranes) in diffusion dialysis for the separation of sulfuric acid and nickel sulphate. In [22], a batch diffusion dialysis equipment that worked in a counter-current mode with two anion-exchange membranes (Fumasep-FAD and Neosepta-AFN) was used to separate acid from a mixture of H_2_SO_4_ and CuSO_4_.

The aim of this communication is to compare three commercial anion-exchange membranes used for the separation of a model mixture of inorganic acids and ferric salts by continuous diffusion dialysis and to establish the basic characteristics of this dialysis process. Model mixtures were selected to approximate the composition of the liquids from stainless steel pickling in inorganic acids [1].

## 2. Theory

**Figure 1:**
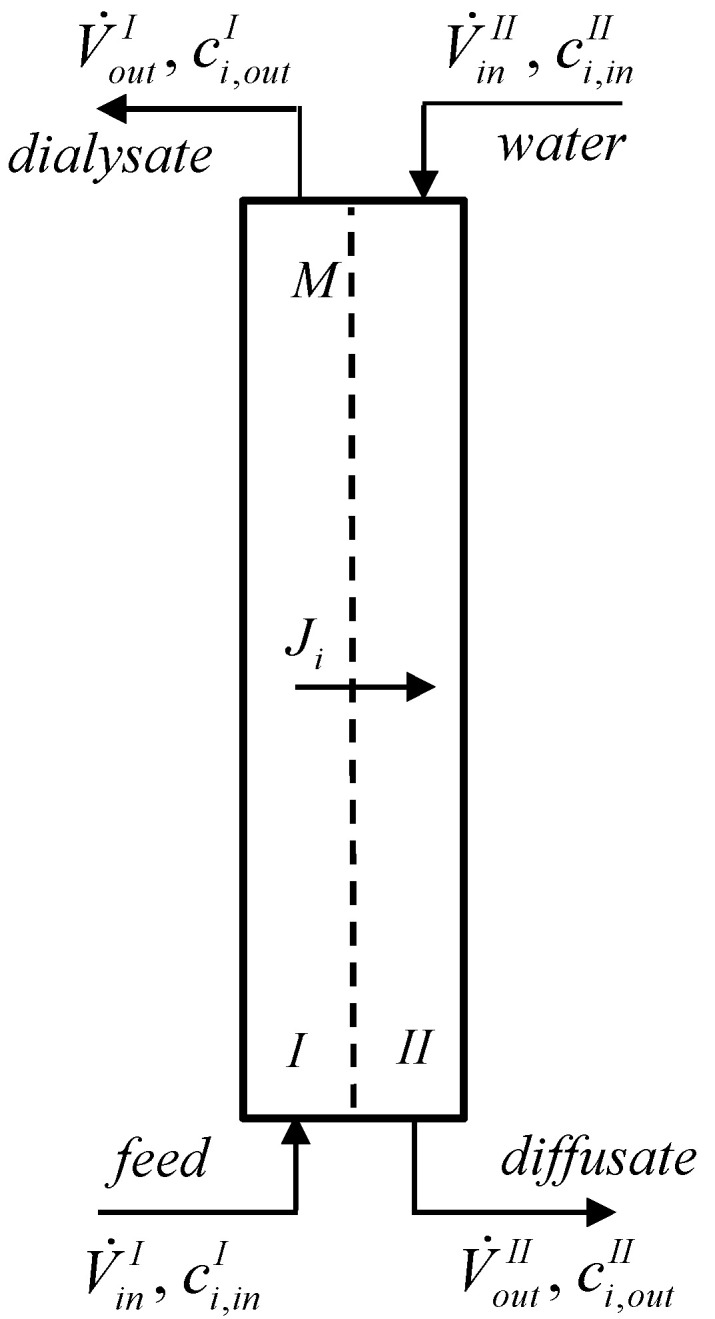
Schematic of the continuous dialyzer: *I*, *II*—compartments, *M*—membrane [15].

A continuous counter-current dialyzer with two compartments separated by an anion-exchange membrane was used to study diffusion dialysis (Figure 1). The feed (mixture of acid and salt) flows into the bottom of compartment I, while the water flows into the top of compartment II.

For a preliminary evaluation of continuous diffusion dialysis at a steady state, the recovery of acid, *ν_i_*, and the rejection of salt, *R_i_*, are used:

(1) $$\nu_{i}=\frac{{\dot{V}}_{out}^{II}c_{i,out}^{II}}{{\dot{V}}_{in}^{I}c_{i,in}^{I}}\times 100\%$$

(2) $$R_{i}=\left(1-\frac{{\dot{V}}_{out}^{II}c_{i,out}^{II}}{{\dot{V}}_{in}^{I}c_{i,in}^{I}}\right)\times 100\%$$

*c_i_* is the molar concentration and $\dot{V}$ is the volumetric flow rate. The superscripts *j* = *I*, *II* mean the compartments, and the subscripts *in* and *out* mean the inlet and outlet. The recovery of H^+^ ions is calculated from Equation (1), while Equation (2) is used to determine the rejection coefficient of Fe^3+^ ions.

If we write the balance of *i* ions on the differential volume of compartments in the steady state, after appropriate arrangements, we get the differential equations that describe the dependence of concentration *i* ions on the length coordinate *z* [23,24].

(3) $$\frac{dc_{i}^{j}}{dz}=-\frac{1}{{\dot{V}}^{j}}\frac{A}{z_{T}}J_{i}-\frac{c_{i}^{j}}{{\dot{V}}^{j}}\frac{d{\dot{V}}^{j}}{dz}\;\;\;\;j=I,II$$

where *A* is the area of the membrane and *z_T_* is the height of the compartment. The flux of component *i* through the membrane, *J_i_*, can be expressed as

(4) $$J_{i}=K_{i}\left(c_{i}^{I}-c_{i}^{II}\right)$$

where *K_i_* is the overall dialysis coefficient.

If we know the concentrations of *i* ions and volumetric flow rates of all streams in the steady state, then we can numerically integrate the set of differential Equations (3). If this step is followed by the appropriate optimization procedure, the overall dialysis coefficient, *K_i_*, can be obtained.

The separation factor *S* is defined as the ratio of the overall dialysis coefficients of the acid and salt (H^+^ ions and Fe^3+^ ions):

(5) $$S=\frac{K_{H^{+}}}{K_{Fe^{3+}}}$$

Water transport through the membrane can be calculated as a change in volumetric liquid flow at the inlet and at the outlet of the dialyzer.

(6) $$J_{w}=\left(\frac{{\dot{V}}_{out}^{I}}{{\dot{V}}_{in}^{I}}-1\right)\times 100\%$$

## 3. Materials and Methods

A continuous flat-plate two-compartment counter-current dialyzer with an anion-exchange membrane was used for the diffusion dialysis research. The transport properties of three different anion-exchange membranes (Neosepta-AFN, Neosepta-AHA, and Fumasep-FAD) were compared. A basic comparison of the properties of the membranes is shown in Table 1, and a more detailed description of the membranes can be found in [25] for the Fumasep-FAD membrane and in [26] for Neosepta membranes.The Ralex AMH-PES and Ralex AMH-PP membranes (Mega a.s., Czech Republic) were not included in the membrane comparison due to their high thickness (750 μm in wet form, which is approximately 5 to 8 times higher than for other membranes) [27] and, therefore, a significantly lower permeability of ions through these Ralex membranes is expected, as shown in [21].

Before the experiments were started with different types of solutions, pre-treatment of the membrane was carried out. The membrane was transferred to the appropriate anionic form (chloride, nitrate, or fluoride) by filling the dialyzer with the solutions of 0.1 mol L^−1^ HCl, HNO_3_, or HF + HNO_3_, respectively, for 24 h. The dialyzer was then thoroughly washed with water.

The dialyzer height was 1 m, the compartment dimensions were 0.92 m × 0.036 m × 0.0011 m, and the compartment volume was 3.6 × 10^−5^ m^3^. The area of the membrane was 331 cm^2^. The detailed scheme of the experimental set-up can be seen elsewhere [24].

The volumetric liquid flow rate values of the measured inlet streams were 2.8 × 10^−8^, 4.2 × 10^−8^, and 5.6 × 10^−8^ m^3^ s^−1^ (100 mL h^−1^, 150 mL h^−1^, and 200 mL h^−1^); therefore, the flow rate values relative to the membrane area were 3.0, 4.5, and 6.0 L h^−1^ m^−2^. The feed flows into the lower part of compartment I, while the distilled water flows into the upper part of compartment II, and the flows were provided by peristaltic pumps. In all experiments, the liquid flow rate of the feed was always equal to that of water. The temperature was kept constant at a value of 25 ± 0.5 °C. The achievement of a steady state (2 to 4 h of dependence on liquid flow rate) was indicated by a constant value of the ion concentration in three successive samples taken from the dialysate and diffusate streams. Then, volumetric flow rates and ion concentrations were determined in all streams (inlet and outlet) [24].

Tested model mixtures of acid and ferric salt were 3 M HCl + 0.5 M FeCl_3_, 3 M HNO_3_ + 0.5 M Fe(NO_3_)_3_, and 3 M HF + 0.7 M Fe(NO_3_)_3_ (that is, iron concentration 30–40 g L^−1^). The afore mentioned model mixture was chosen to approximate the composition of the solution from the pickling of stainless steel in the hydrofluoric and nitric acid mixtures [15]. In addition to the dialysis experiments with mixtures of acids and their iron salts, experiments with 3 mol L^−1^ acids (HCl, HNO_3_, and HF) were also carried out.

The concentration of Fe^3+^ ions was determined by optical emission spectroscopy with inductively coupled plasma (Integra 6000 ICP-OES, GBC Scientific Equipment, Dandenong, Australia), and the concentration of H^+^ ions was determined by titration with a standard NaOH solution with the counting of precipitation of Fe^3+^ hydroxide.

## 4. Results and Discussion

### 4.1. Recovery Yield of Acid

The recovery of H^+^ ions was calculated according to Equation (1) and is shown in Table 2 for the solutions tested (that is, for acids alone and for mixtures of acid and ferric salts) for a volumetric flow rate of 100–200 mL h^−1^.

**Figure 2:**
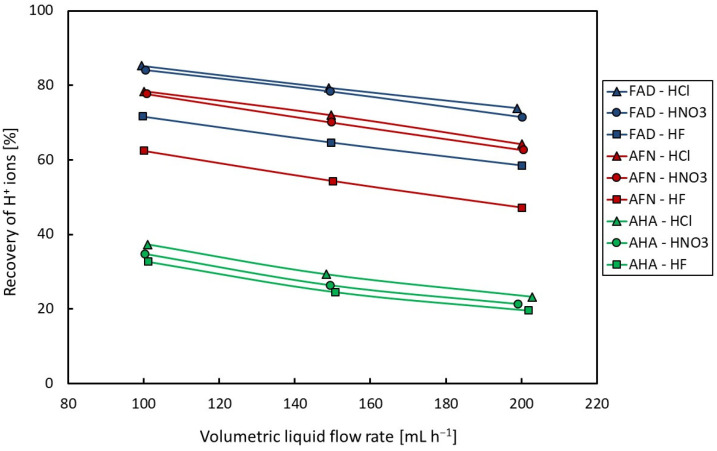
Dependence of recovery of H^+^ ions on volumetric flow rate (for acids).

**Figure 3:**
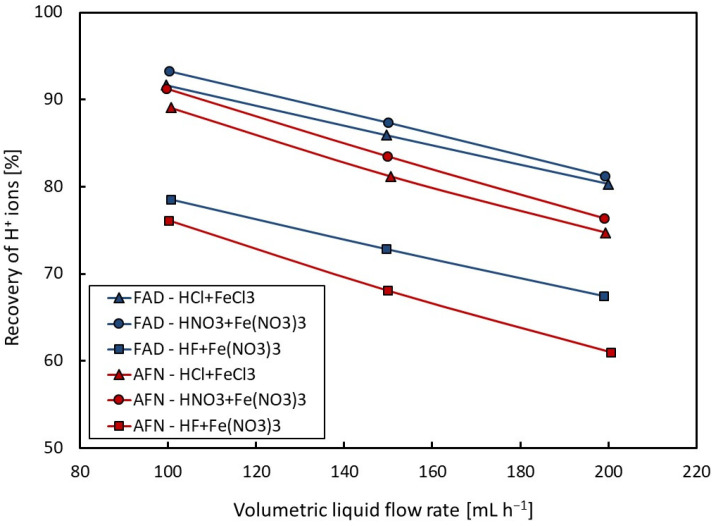
Dependence of recovery of H^+^ ions on volumetric flow rate (for mixture of acid and Fe^3+^ salt).

The dependencies of the recovery of H^+^ ions on the volumetric flow rate are presented in Figure 2 for acids alone and in Figure 3 for the mixture of acid and ferric salt. From Figure 2 and Figure 3, it is apparent that acid recovery is strongly affected by the volumetric flow rate. A decrease in the recovery of H^+^ ions can be observed with an increasing volumetric flow rate for all tested solutions due to a decrease in the mean dwell time of the liquid in the dialyzer.

A comparison of the results obtained when acid alone is tested shows that the values of acid recovery of hydrochloric and nitric acids are comparable, while somewhat lower values were found for hydrofluoric acid. It is further evident that the Fumasep-FAD membrane shows slightly higher values of acid recovery compared to those of the Neosepta-AFN membrane. This is probably due to the slightly lower thickness of the Fumasep-FAD membrane (see Table 1). The acid recovery values found for the Neosepta-AHA membrane were significantly lower, from 20% to 37% in the test range of the volumetric flow rate (100–200 mL h^−1^). These values of acid recovery are insufficient for the adequate separation of acids and salts; therefore, the Neosepta-AHA membrane was excluded from further testing of the diffusion dialysis of a mixture of acids and their ferric salts. The reason is probably the higher thickness and higher resistance to pH of this membrane (see Table 1).

From Table 2 and from Figure 3 in which the values of recovery of H^+^ ions are shown for the Neosepta-AFN and Fumasep-FAD membranes, it is evident that for all mixtures tested, the Fumasep-FAD membrane shows higher acid recovery values than the Neosepta-AFN membrane. It was also found that the presence of ferric ions improves the transport of H^+^ ions through the membrane, i.e., it increases the recovery yield of acid. The recovery yield of nitric acid was also slightly higher than that of hydrochloric acid for the mixtures with ferric salt.

### 4.2. Rejection Coefficient of Iron

**Figure 4:**
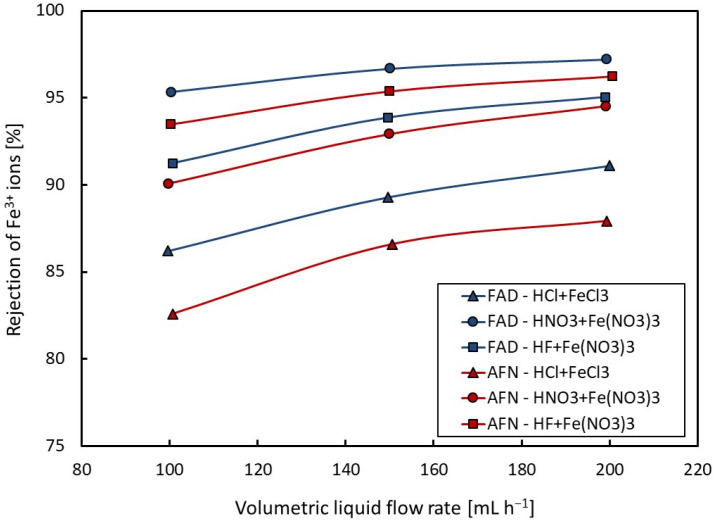
Dependence of rejection of Fe^3+^ ions on volumetric flow rate.

The values of the rejection of Fe^3+^ ions were determined from Equation (2) and are summarised in Table 3. The dependencies of the rejection coefficient on volumetric flow rate are shown in Figure 4 for the acid and ferric salt model mixture and for the Fumasep-FAD and Neosepta-AFN membranes.

The rejection coefficients of ferric ions increase with an increase in the volumetric flow rate, which is due to the decrease in the mean dwell time of the liquid in the dialyzer, as expected. The lowest values of rejection of Fe^3+^ ions were found to be for the HCl + FeCl_3_ mixture; on the contrary, the presence of nitrate anions increased the rejection of ferric ions. The Fumasep-FAD membrane shows higher rejection values than the Neosepta-AFN membrane; the exception was the HF + Fe(NO_3_)_3_ mixture.

### 4.3. Overall Dialysis Coefficients

The overall dialysis coefficient was determined from the concentration and volumetric flow rates values at a steady state by a numerical integration of the set of Equation (3), where *J_i_* is expressed by Equation (4). The integration of a set of Equation (3) was performed in both directions of the longitudinal coordinate *z* [23]. The calculated values of the overall dialysis coefficients of H^+^ ions are shown in Table 4, and those of Fe^3+^ ions are shown in Table 5 for the tested range of volumetric flow rate from 100 to 200 mL h^−1^.

The values of the overall dialysis coefficients of H^+^ ions correspond to the results of the recovery of H^+^ ions mentioned in Section 4.1. The highest values were found for the Fumasep-FAD membrane, slightly lower for the Neosepta-AFN membrane, and for the Neosepta-AHA mebrane, the values of K_H+_ were approximately five times lower. The reason is probably the thickness of the membranes (see Table 1). The Fumasep-FAD membrane has the smallest thickness; on the contrary, the Neosepta-AHA membrane has the highest one.

In Table 4, it can be further seen that the values of K_H+_ for the mixture of acid and ferric salt were always higher than the values for the acid alone, i.e., the ferric salts facilitate the transport of acid through the membrane. The highest acid recovery values of the overall dialysis coefficient of H^+^ ions were found for the mixture of 3 M HNO_3_ + 0.5 M Fe(NO_3_)_3_ and the Fumasep-FAD membrane.

The measured K_H+_ values for hydrochloric acid and the Neosepta-AFN membrane correspond to those given in the literature [23] where diffusion dialysis of hydrochloric and phosphoric acids are compared. For 3 M H_3_PO_4_, the value of the overall dialysis coefficient of the acid is approximately 0.2 × 10^−6^ m/s (for the volumetric liquid flow rate 100 mL h^−1^), which is ten times lower than for hydrochloric and nitric acids. Furthermore, it is shown here that the permeability of the Neosepta-AFN membrane decreases with increasing acid concentration for phosphoric acid; on the contrary, it increases with increasing acid concentration for hydrochloric acid [23].

It can be seen in Table 5 that the overall dialysis coefficient for ferric ions is about one to two orders of magnitude lower than that for the H^+^ ions. The Fumasep-FAD membrane showed lower values of K_Fe3+_ than the Neosepta-AFN membrane; the exception was the mixture of HF + Fe(NO_3_)_3_. The highest values of the overall dialysis coefficient of Fe^3+^ were found for the mixture of HCl + FeCl_3_; in contrast, in the presence of nitrates, the values of K_Fe3+_ were lower.

The observed results of the membrane comparison also correspond to the results of [22], where a batch diffusion dialysis with two anion-exchange membranes (Fumasep-FAD and Neosepta-AFN) was used to separate the acid from the mixture of H_2_SO_4_ and CuSO_4_. It was found there that the permeabilities for the acid and water were higher for the Fumasep-FAD membrane than for Neosepta-AFN. The permeability of the Fumasep FAD membrane for Cu^2+^ ion was also slightly higher; therefore, the rejection of Cu^2+^ was also slightly lower than that of Neosepta-AFN.

### 4.4. Separation Factor

The separation factor was calculated as the ratio of the overall dialysis coefficients of H^+^ ions and Fe^3+^ ions using Equation (5). The values of the separation factors are summarised in Table 6 for the tested range of the volumetric liquid flow rate from 100 to 200 mL h^−1^.

It is evident from Table 6 that the Fumasep-FAD membrane showed higher separation factor values than the Neosepta-AFN membrane for all mixtures tested. It was further found that both membranes tested had better separation properties for the mixture of HNO_3_ + Fe(NO_3_)_3_ than for the mixtures of HCl + FeCl_3_ and 3 M HF + 0.7 M Fe(NO_3_)_3_ for which the separation factor values were comparable.

For both membranes tested, a phenomenon described in the literature [15] was found, that is, the recovery yield of nitrates was much higher than the recovery yield of fluorides. The same applies to the values of the overall dialysis coefficients. Also, the separation factor values for nitrates/ferric ions reached higher values (approximately 4×) than the separation factors of fluorides/ferric ions. The reason is that in the mixture of HNO_3_, HF, and a ferric salt, the FeF^2+^ complex predominates, and this divalent cation practically does not pass through the anion exchange membrane. Therefore, nitric acid passes through the membrane faster than hydrofluoric acid. In some cases, the amount of nitric acid in the diffusate can be higher than in the feed [15].

### 4.5. Water Transport through the Membrane

Due to the transport of water through the membrane, there were changes in the volumetric liquid flow rates at the inlet and outlet. This means that while the inlet streams of the feed and water were the same, the outlet streams (dialysate and diffusate) were slightly different. At the same time, the volume balance of all streams was within 0.2% for all measurements. The water flow through the membrane was determined using Equation (6), and its values are shown in Table 7 for a volumetric flow rate of 150 mL h^−1^. The values of water transport were almost independent of flow rate.

For most of the solutions tested, there was a flow of water from compartment I to compartment II; thus, the amount of the dialysate (and also the concentration of components) decreased, and that of the diffusate increased. Only in the case of the tested mixture of 3 M HF and 0.7 M Fe(NO_3_)_3_ and the Fumasep-FAD membrane was the flow of water through the membrane the opposite, that is, from compartment II to compartment I. Higher values of water transport were observed for acids alone (the highest for 3 M HNO_3_) than for mixtures of acids and Fe^3+^ salt where there was no significant influence on the concentrations in the dialysate and diffusate.

The observed results correspond to the results of [22] that aimed to separate the H_2_SO_4_ and CuSO_4_ mixture by diffusion dialysis. It was found there that for H_2_SO_4_ solutions, water flux was observed from the dialysate to the diffusate for all concentrations investigated. On the contrary, the presence of CuSO_4_ (higher concentration) caused the reverse flow of water (from diffusate to dialysate). Determining the flow of water through the membrane and its direction during the diffusion dialysis of mixtures of acids and salts is a complex problem that depends on the composition of the solutions, the type of membrane, and the flow rate, and there is not enough information on it in the literature.

## 5. Conclusions

The continuous diffusion dialysis of a model mixture of acids (HCl, HNO_3_, and HF) and their ferric salts was investigated in a counter-current dialyzer with three different anion-exchange membranes (Fumasep-FAD, Neosepta-AFN, and Neosepta-AHA). From steady-state measurements, the basic transport characteristics of diffusion dialysis, acid recovery, and metal rejection, and their dependences on volumetric flow rates, were evaluated. The dialysis coefficients of the acids and iron and the acid/iron separation factors were also determined. In the volumetric flow rate test range from 100 to 200 mL h^−1^ (from 3 to 6 L h^−1^ m^−2^, relative to the membrane area), the Fumasep-FAD membrane was found to show slightly higher values of acid recovery and overall acid dialysis coefficient than the Neosepta-AFN membrane. In contrast, the values found for the Neosepta-AHA membrane were significantly lower, and they were insufficient for the adequate separation of acids and iron salts. A comparison of the diffusion dialysis results for acid and iron salt mixtures showed better results for acid recovery, iron rejection, and acid/iron separation factors for the Fumasep-FAD membrane than for the Neosepta-AFN membrane. A similar behaviour of both of these membranes depending on the volume flow of the liquid and the properties of the tested substances was also observed, i.e., the acid recovery decreased with an increase in the volumetric flow rate, whereas the iron rejection increased with an increase in the volumetric flow rate. It was also found that the presence of ferric ions improves the transport of H^+^ through the membrane, i.e., it increases the acid recovery and overall dialysis coefficient of acids. Furthermore, it was validated that the values of the separation factor acid/iron were significantly higher for the mixture HF + Fe(NO_3_)_3_ than for the mixture HCl + FeCl_3_. The results obtained showed that both of these anion-exchange membranes (Fumasep-FAD and Neosepta-AFN) can be considered good separators for the tested mixtures of inorganic acid and iron salt.

## Figures and Tables

**Table 1:** Properties of the tested membranes.

Membrane	Fumasep-FAD	Neosepta-AFN	Neosepta-AHA
Producer	Fumatech BWT GmbH (Bietigheim-Bissingen, Germany)	Astom Corp. (Tokyo, Japan)	Astom Corp. (Tokyo, Japan)
Counter ion	Bromide	Chloride	Chloride
Thickeness (wet)	100 μm	160 μm	220 μm
pH stability	0–8	0–8	0–14
Temperature (°C)	15–40	≤40	≤40
Electric resistence (Ω cm^2^)	1.2	2.6	4.1
Delivery form	dry	wet	Wet

**Table 2:** Recovery of H^+^ ions.

	Fumasep-FAD			Neosepta-AFN			Neosepta-AHA		
Flow rate (mL h^−1^)	100	150	200	100	150	200	100	150	200
3 M HCl	85%	79%	74%	78%	72%	64%	37%	29%	23%
3 M HNO_3_	84%	78%	71%	78%	70%	63%	35%	26%	21%
3 M HF	72%	65%	58%	62%	54%	47%	33%	25%	20%
3 M HCl + 0.5 M FeCl_3_	92%	86%	80%	89%	81%	75%			
3 M HNO_3_ + 0.5 M Fe(NO_3_)_3_	93%	87%	81%	91%	83%	76%			
3 M HF + 0.7 M Fe(NO_3_)_3_	79%	73%	67%	76%	68%	61%			

**Table 3:** Rejection of Fe^3+^ ions.

	Fumasep-FAD			Neosepta-AFN		
Flow rate (mL h^−1^)	100	150	200	100	150	200
3 M HCl + 0.5 M FeCl_3_	86%	89%	91%	83%	87%	88%
3 M HNO_3_ + 0.5 M Fe(NO_3_)_3_	95%	97%	97%	90%	93%	95%
3 M HF + 0.7 M Fe(NO_3_)_3_	91%	94%	95%	93%	95%	96%

**Table 4:** Overall dialysis coefficient of H^+^ ions (K_H+_ × 10^6^ m/s).

	Fumasep-FAD			Neosepta-AFN			Neosepta-AHA		
Flow rate (mL h^−1^)	100	150	200	100	150	200	100	150	200
3 M HCl	3.8	4.0	4.1	2.6	2.7	2.8	0.49	0.51	0.51
3 M HNO_3_	3.2	3.4	3.4	2.4	2.5	2.5	0.44	0.44	0.45
3 M HF	2.0	2.2	2.2	1.3	1.4	1.5	0.40	0.41	0.42
3 M HCl + 0.5 M FeCl_3_	9.2	7.7	6.3	5.9	4.8	4.7			
3 M HNO_3_ + 0.5 M Fe(NO_3_)_3_	11.2	8.0	6.8	7.3	5.4	4.9			
3 M HF + 0.7 M Fe(NO_3_)_3_	4.5	3.8	3.6	2.6	2.6	2.5			

**Table 5:** Overall dialysis coefficient of Fe^3+^ (K_Fe3+_ × 10^7^ m/s).

	Fumasep-FAD			Neosepta-AFN		
Flow rate (mL h^−1^)	100	150	200	100	150	200
3 M HCl + 0.5 M FeCl_3_	1.5	1.6	1.7	1.8	2.0	2.1
3 M HNO_3_ + 0.5 M Fe(NO_3_)_3_	0.39	0.41	0.43	0.94	1.0	1.0
3 M HF + 0.7 M Fe(NO_3_)_3_	0.86	0.86	0.88	0.63	0.65	0.7

**Table 6:** Separation factor (H^+^/Fe^3+^).

	Fumasep-FAD			Neosepta-AFN		
Flow rate (mL h^−1^)	100	150	200	100	150	200
3 M HCl + 0.5 M FeCl_3_	63	47	38	33	24	22
3 M HNO_3_ + 0.5 M Fe(NO_3_)_3_	285	194	157	77	54	48
3 M HF + 0.7 M Fe(NO_3_)_3_	52	46	40	42	39	36

**Table 7:** Water transport through the membrane (for flow rate of 150 mL h^−1^).

	Fumasep-FAD	Neosepta-AFN	Neosepta-AHA
3 M HCl	−10%	−8%	−2%
3 M HNO_3_	−13%	−10%	−4%
3 M HF	−3%	−4%	−2%
3 M HCl + 0.5 M FeCl_3_	−2%	−5%	
3 M HNO_3_ + 0.5 M Fe(NO_3_)_3_	−1%	−5%	
3 M HF + 0.7 M Fe(NO_3_)_3_	7%	−2%	

## Data Availability

Data is contained within the article.

## Nomenclature

**Symbols**	
*A*	membrane area, m^2^
*C*	concentration, mol L^−1^ (M)
*J*	molar flux, kmol m^−2^ s^−1^
*J*_w_	water transport through the membrane, %
*K*	overall dialysis coefficient, m s^−1^
*R*	rejection, %
*S*	separation factor, −
$\dot{V}$	volumetric flow rate, m^3^ s^−1^ (mL h^−1^)
*ν*	recovery, %
z	length, m
**Subscripts and Superscripts**	
*I*	related to compartment I
*II*	related to compartment II
*i*	related to *i* ion
*in*	inlet
*out*	outlet
